# Mating with Multi-Armed Bandits: Reinforcement Learning Models of Human Mate Search

**DOI:** 10.1162/opmi_a_00156

**Published:** 2024-08-15

**Authors:** Daniel Conroy-Beam

**Affiliations:** Department of Psychological and Brain Sciences, University of California, Santa Barbara

**Keywords:** mate search, mate choice, reinforcement learning, agent-based modeling, Thompson sampling

## Abstract

Mate choice requires navigating an exploration-exploitation trade-off. Successful mate choice requires choosing partners who have preferred qualities; but time spent determining one partner’s qualities could have been spent exploring for potentially superior alternatives. Here I argue that this dilemma can be modeled in a reinforcement learning framework as a multi-armed bandit problem. Moreover, using agent-based models and a sample of *k* = 522 real-world romantic dyads, I show that a reciprocity-weighted Thompson sampling algorithm performs well both in guiding mate search in noisy search environments and in reproducing the mate choices of real-world participants. These results provide a formal model of the understudied psychology of human mate search. They additionally offer implications for our understanding of person perception and mate choice.

## INTRODUCTION

Mate choice poses a judgment under uncertainty problem. Most of the characteristics people prefer in potential partners require time and effort to accurately observe. But time spent learning about one partner is time that could have been spent pursuing others. Successful mate choice therefore requires balancing effort between investigating specific partners and exploring the broader market in search of potentially superior alternatives. This core problem of human mate choice has been surprisingly neglected by the otherwise large literature on human mating (although see Miller & Todd, 1998). Here I argue that a framework from the reinforcement learning literature, multi-armed bandit models, is well-suited to modeling how people navigate this fundamental decision problem of mate choice.

Most research on human mate selection has been guided by a core premise: successful mate selection requires pursuing partners who possess preferred qualities (e.g., kindness, health, physical attractiveness, wealth, intelligence, etc.; Buss, 1989; Fletcher & Simpson, 2000). Most research has focused on these preferred qualities themselves. This includes determining what qualities people find desirable (Buss, 1989; Fletcher & Simpson, 2000; Hill, 1945; Walter et al., 2020), measuring how people esteem partners who possess these preferred qualities (Eastwick & Finkel, 2008; Gerlach et al., 2019; Li et al., 2013), and, to a lesser extent, modeling how people navigate among alternative partners who are known to differ in preferred characteristics (Conroy-Beam, 2021; French & Kus, 2008; Kalick & Hamilton, 1986; Miller & Todd, 1998; Smaldino & Schank, 2012; Xie et al., 2015).

Surprisingly neglected in this literature is the question of how people come to know which partners possess the characteristics they desire. Most studies of mate choice assume (either implicitly or explicitly) that people can immediately perceive the ground truth features of their potential mates. However, in real life, information is not free. Out of a set of potential mates, one may be able to quickly surmise which potential mates are relatively physically attractive (Olson & Marshuetz, 2005). But knowing which have a good sense of humor requires at least a conversation. Discerning personality with high confidence requires observing a person over time and, ideally, across a range of contexts (Buss & Craik, 1983). You may not know on whom you can count on in a crisis until a crisis occurs. Impressions of personality or relationships-related variables at initial acquaintance have non-zero but modest accuracy, with correlations between judgments and criterion measurements generally up to *r* = .30 at best, and generally lower (Ambady et al., 2000; Gangestad et al., 1992). These first impressions do of course importantly shape future interactions (Li et al., 2013; Shteingart et al., 2013). Nonetheless, impressions are more accurate for targets with whom we are better acquainted (Funder & Colvin, 1988). This means that knowing whether a potential partner possesses your preferred characteristics requires investing time toward forming an accurate and informed impression of them.

Information is also inherently noisy. Even relatively “objective” variables such as a person’s physical attractiveness can vary across contexts depending on, for example, appearance enhancement efforts (Kowal et al., 2022; Osborn, 1996). Personality traits have been modeled as reaction norms: person-specific functions mapping context to behavior, which allow variation around environmentally contingent means (Denissen & Penke, 2008; Dingemanse et al., 2010; Sih et al., 2004). Whereas the human mating literature has largely assumed that potential mates have true features that can be accurately described as static, scalar values, it is more realistic to think of potential mates’ features in terms of probability distributions described by parameters that can be estimated only through repeated sampling.

Exacerbating these problems, search requires the expenditure of finite resources. There’s only so much time in a week available to go on dates, only so much money to spend on coffee, outings, or romantic gifts, and so on. And critically, resources spent doing reconnaissance on one partner could always have been spent searching other partners—some of whom might have turned out to be superior options.

Because of these limitations, mate search poses a critical decision problem: how should you decide which partners to pursue when faced with uncertain options, where any interaction gives only an imperfect picture of a potential mate, and search resources are finite? This question has been largely neglected by the human mating and relationships literatures but its answer is essential to understanding human mate choice. Fortunately, reinforcement learning provides an ideal framework for modeling how people solve this decision problem.

Reinforcement learning models scenarios where decision-making agents must choose among actions of uncertain reward value (Sutton & Barto, 2018). Central to this decision is the notion of an exploitation-exploration trade-off. Maximizing reward requires repeatedly choosing the most rewarding actions—“exploitation.” However, discovering which actions are rewarding requires “exploration:” testing different actions in order to learn which actions are most rewarding. Maximizing reward requires a balance of these two goals: exploring enough to learn which actions are rewarding, but leaving enough time for exploitation to reap the value of one’s knowledge.

One popular class of reinforcement learning models—multi-armed bandit models—are particularly well suited to modeling the problem of mate search. Multi-armed bandit models imagine an agent playing a slot machine with multiple arms. Each arm has a payout rate that is uncertain to the agent and observable only through repeated pulls. However, the agent has only finite opportunities to pull the arms. The agent must allocate its pulls such that it explores enough to learn which arms are the most rewarding but also exploits those arms that turn out to be relatively rewarding.

Multi-armed bandit models closely mimic the problem of mate search. Here, the mate seeker is the decision-making agent and each potential mate is an arm of a multi-armed bandit. Each potential mate has an unknown true “mate value”—that is how rewarding they would be if secured as a romantic partner (Conroy-Beam et al., 2022; Sugiyama, 2015). Pursuing a given mate for some amount of time (i.e., pulling their arm of the multi-armed bandit) will reveal some noisy information about their true mate value. However, time available for pursuing potential mates is finite. Mate seekers thus must allocate their resources so as to balance the classic exploitation-exploration trade-off: they must explore their potential mates enough to learn which are relatively high in mate value but must retain enough resources to successfully pursue those mates that they learn are highest in value to them.

Fortunately, the reinforcement learning literature offers several algorithms which are good solutions to this multi-armed bandit problem. One simple but effective solution to multi-armed bandit problems is the *ε*-greedy algorithm. Most of the time, this algorithm pulls whichever arm of the bandit appears most rewarding in the moment—a “greedy,” exploitative action. However, on a small but random number of trials—dictated by a parameter, *e*—the algorithm explores by pulling a random arm. These exploratory trials allow the agent to learn which arms are most rewarding whereas the exploitative trials ensure that the agent achieves high reward.

The *ε*-greedy algorithm can perform well, but its simplicity does have a cost. Chiefly, *ε*-greedy explores its options randomly, without consideration of the knowledge state of the agent. Algorithms that explore intelligently in light of the agent’s current uncertainty can outperform *ε*-greedy. One such algorithm is the upper confidence bound algorithm (*UCB*). On each decision trial, this algorithm pulls the bandit arm that according to the function: *A*_*t*_ = arg max_*a*_{*Q*(*a*) + *c* * $\sqrt{\ln t/N\left(a\right)}$}, where *A*_*t*_ is the arm chosen at time *t*, *Q*(*a*) is the estimated reward of arm *a*, *c* is a constant parameter that controls the agent’s balance of exploration vs. exploitation, *t* is the total number of decision trials the agent has completed, and *N*(*a*) is the number of decision trials in which the agent previously chose to pull arm *a* (Sutton & Barto, 2018). All else equal, this algorithm favors choosing the arm that has previously paid out the highest reward. However, each time a given arm is not chosen by the agent, the square root term for that arm increases and so does the likelihood this arm will be chosen in the future. In essence, this algorithm favors occasionally testing arms that have been infrequently chosen and whose true reward value is therefore most uncertain. Unlike the *ε*-greedy algorithm, this algorithm balances exploration and exploitation strategically by selectively exploring specific arms when uncertainty about their true value is great enough that their true value could potentially exceed the known value of more preferred arms.

Thompson sampling, a Bayesian algorithm, also balances exploration and exploitation strategically but in a slightly different way from UCB. Thompson sampling agents maintain a prior distribution over the reward value of each bandit arm. On each decision trial, each agent samples a reward value from their prior distribution for each arm and pulls the arm with the highest sampled reward. The agent then uses the reward received from this pull to update its prior in Bayesian fashion. Consequently, this algorithm initially explores a lot when priors are uninformed and wide, but gradually shifts to exploitation as the agent learns which arms are the most rewarding.

Do these models approximate how people solve the exploitation-exploration dilemma at the heart of mate search? Here, I answer this question by applying reinforcement learning models in agent-based modeling framework called “couple simulation.” This approach represents members of real-world couples as agents in simulated mating markets and models these agents choosing one another as mates according to alternative models of mate choice. The core question of couple simulation is: what models of choice are capable of accurately reproducing the mate choice decisions of real-world participants? Prior simulation studies have shown that those models that most closely approximate a population’s true decision making model tend to most accurately reproduce couples sampled from that population (Conroy-Beam, 2021; Conroy-Beam et al., 2022). Couple simulation therefore provides a useful framework for empirically testing the utility of reinforcement learning models as models of human mate search.

To assess the performance of reinforcement learning models as models of mate search, I first used agent-based models to explore whether these models can indeed solve the exploration/exploitation dilemma inherent to real mate search. These initial models demonstrated that while several reinforcement learning models can perform well in guiding human mate search, a Thompson sampling model worked best of all. I then used couple simulation to show that a Thompson sampling model performs well in reproducing real-world mate choices even under conditions of very noisy information. These results indicate that human mate search can indeed be successfully modeled as a reinforcement learning problem.

## MODELING

In order to empirically test the suitability of reinforcement learning models as models of human mate search, I first had to establish two things. First, because reinforcement learning models have not been previously applied to human mate choice, I first sought to establish that reinforcement learning algorithms can indeed successfully guide mate search under conditions of imperfect information. Second, I sought to establish that the mate choices made by a reinforcement learning mate search algorithm would indeed be reproducible in a couple simulation framework despite their inherently probabilistic nature. Here I present two agent-based models that demonstrate that reinforcement learning algorithms can solve the problem of mate search and do make reproducible mate choices.

### Reinforcement Learning Algorithms as Models of Mate Search

**Figure 1.**
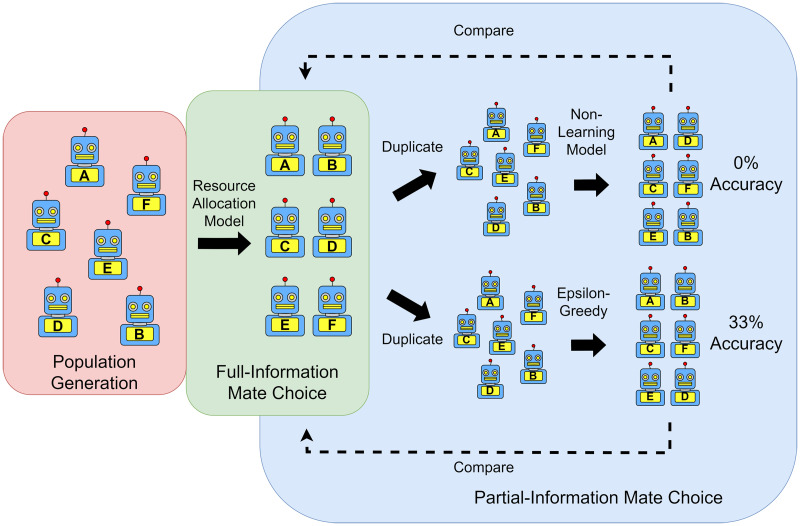
**Diagram of reinforcement learning evaluation process.** *Note*. A diagram of the modeling process for evaluating suitability of reinforcement learning models for navigating mate search. I compared mate choices made by reinforcement learning models under conditions of noisy information to an established mate choice model under condition of full information. Models were compared on simulation accuracy (percent full-information couples reproduced). Only two models are displayed in the figure for space reasons, however seven total models were compared.

To identify models capable of accomplishing mate choice under incomplete information, I tested the ability of several reinforcement learning models to approximate full-information mate choice. For this, I simulated a population of agents based on real human data and had these agents select one another as mates in a full-information environment using a mate choice model that has been previously shown to perform well on human data. I then simulated mate choice in this population again under conditions of imperfect information, using either a control mate choice model with no reinforcement learning or one of six reinforcement learning models. I finally tested the ability of these models to reproduce the choices made by the full-information model. Figure 1 diagrams this process.

#### Generating Agent Population.

I generated a population of *N* = 500 agents following the procedure outlined in Conroy-Beam (2021). Agents were based on a sample of *n* = 1,044 participants who were members of *k* = 522 committed, romantic dyads. Participants in this sample reported their ideal mate preferences as well as their own traits on 16 dimensions (see Human Data for details). These ratings were used to create the agent population in a three-step process: (1) the model resampled from each dimension up to an *N* of 500, (2) the matrix of resampled values was multiplied by the Cholesky decomposition of the human data’s correlation matrix, and (3) the resulting trait and preference dimensions were rescaled to share the mean and standard deviation of the original human variables. Half of agents were generated to be male and the other half were female; male and female agents were generated separately using human data from the relevant sex. This yields a sample of simulated agents who have traits and preferences whose distributions and covariance matrices mimic those found in real-world participants.

#### Full Information Mate Choice.

After generation, agents selected each other as mates in a full-information context. Here, agents were able to perfectly perceive the traits of each of their potential mates. Mate choice followed the resource allocation model, because this has separately been shown to best approximate human mate choice among a range of alternative models (Conroy-Beam, 2021). In this model, each agent possesses finite set of resources that they allocate to potential mates. Agents initially allocate resources across potential mates directly in proportion to mate value. Following previous research, mate value is computed as proportional to the Euclidean distance between the agent’s preference vector and each potential mate’s trait vector, rescaled such that higher values indicated a shorter distance (Conroy-Beam et al., 2022). Across a series of 50 steps, agents next iteratively re-allocate their resources across potential mates in proportion to the resources sent and received from each potential mate in the prior allocation step. At the end of this reallocation phase, agents typically converge on one potential mate with whom they have high mutual investment. Agents are then paired with the potential mate in whom they are most invested if that agent is also most invested in them in return (ties broken randomly). Agents failed to pair if the partner in whom they were most invested did not reciprocally invest the plurality of their resources in them; these agents were marked as single. This resulted in *k* = 215 agent couples and *n* = 70 single agents that were used as the reference population for evaluating the performance of reinforcement learning models of mate choice.

#### Partial Information Mate Choice.

To evaluate the performance of the reinforcement learning models, the same population of agents selected mates again under a condition of partial information. Here, agents progressed through 10,000 learning trials. On each learning trial, the agent could pursue one potential mate. Upon pursuit, agents received an observation of that potential mate’s true mate value drawn from a normal distribution centered on the potential mate’s true mate value. The standard deviation of this normal distribution was scaled such that, across agents, 50% of the variance in perceptions of potential mates was attributable to random noise.

Agents selected mates in this environment using one of seven different mate search models: a non-learning baseline model, an epsilon-greedy model, an upper confidence bound model, a Thompson sampling model, or reciprocity-weighted versions of the epsilon-greedy, upper confidence bound, and Thompson sampling models.

##### *Non-Learning*.

As a baseline model, non-learning agents pursued potential mates with no attempt to learn their true mate values. In this model, agents made a single observation of each potential mate and then proceeded through resource allocation mate choice exactly as in the full-information model.

##### *Mate-Value-as-Reward Models*.

In the three mate-value-as-reward models, agents attempted to maximize the mate value of pursued potential mates by allocating their observation opportunities using either an *ε*-greedy, upper confidence bound (UCB), or Thompson sampling algorithm. In all models, agents initially assumed all potential mates were maximal in mate value (i.e., *mv* = 10 for all agents). In the *ε*-greedy model, agents observed the potential mate with the highest learned mate value on each trial with probability 1-*ε* (breaking ties randomly) and observed a random potential mate on each trial with probability *ε*. Observations were used to update the agent’s perception of the mate’s mate value using a simple averaging method.

UCB agents chose to observe the potential mate according to the function *M*_*t*_ = arg max_*m*_{*mv*(*m*) + *c* * $\sqrt{\ln t/N\left(m\right)}$}, where *M*_*t*_ is the mate observed at time *t*, *mv*(*m*) is the learned mate value of potential mate *m*, *c* is an exploration constant, *t* is the total number of learning trials the agent has completed, and *N*(*m*) is the number of times mate *m* has been observed so far. Agents again updated mate value perceptions of observed mates using a simple average.

Thompson sampling agents had a normally distributed prior distribution over the mate value of each potential mate, initially centered for each mate on *M* = 10. At each time step, these agents drew a mate value belief randomly from each mate’s prior distribution and observed the potential mate with the highest mate value belief. This observation was used to update the agent’s prior distribution for that mate according to Bayes’ rule.

The *ε* parameter for the epsilon greedy model, the *c* parameter for the UCB model, and the initial *SD* of the Thompson sampling agents were set to *ε* = .2, *c* = 1.5, and *SD* = .2 based on an initial parameter tuning. Parameter tuning was accomplished by running the model comparison approach on a smaller agent population across a wide range of parameter settings for each model and selecting the parameter value that maximized performance for each model.

##### *Reciprocity-Weighted Reward Models*.

Each of the mate-value-as-reward models will over time learn which potential mates are highest in mate value and direct pursuit toward these high-value partners. However, because mate choice is mutual, successful mate choice requires more than just pursuing the highest mate value partners—rather, one must pursue the highest mate value partners that are willing to pursue you in return. In attempt to encourage more strategic mate pursuit, I created three reciprocity-weighted reward models. These were nearly identical to the mate-value-as-reward models. But rather than pursuing just the highest mate value potential mates, agents in the reciprocity-weighted reward models allocated observations following a reward function weighted by reciprocity of pursuit between the agent and their potential mates.

To compute reciprocity for potential mate *i*, agent *j* computed *reciprocity*_*i*_ = obsi,jobsj,i, where *obs*_*i*,*j*_ was the number of times potential mate *i* pursued agent *j* and *obs*_*j*,*i*_ was the number of times agent *j* pursued potential mate *i*. This reciprocity value was multiplied by the mate value learned for that potential mate in the reciprocity-weighted *ε*-greedy model (RWEG), by the summed reward estimate in the reciprocity-weighted UCB model (RWUCB), and by the agent’s sampled mate value belief in the reciprocity-weighted Thompson sampling model (RWTS). In this way, agents were more likely to observe potential mates who were both (1) higher in learned mate value and (2) who were showing reciprocated interest in the agent. If the agent *j* was choosing a potential mate *i* frequently but *i* was not pursuing *j* in return, this would decrease the perceived reward value of that potential mate and make the agent more likely to pursue other, more reciprocally interested partners. The *ε*, *c*, and Thompson sampling *SD* parameters were set to .15, .2, and .25 respectively based on initial parameter tuning.

For all models, observations were allocated in blocks of 1,000 up to 10,000 total observations. At the end of each block, each agent provisionally paired with the potential mate that they had pursued the most up to that point provided this mate had also allocated the plurality of their observations to the agent in return. Splitting allocations into blocks allowed assessment of which models best approximate the full-information model as well as how quickly the models converge on the full-information model’s choices. Furthermore, each model was repeated 10 times. Here I report the average performance of each model in each observation block across its 10 iterations.

#### Results.

**Figure 2.**
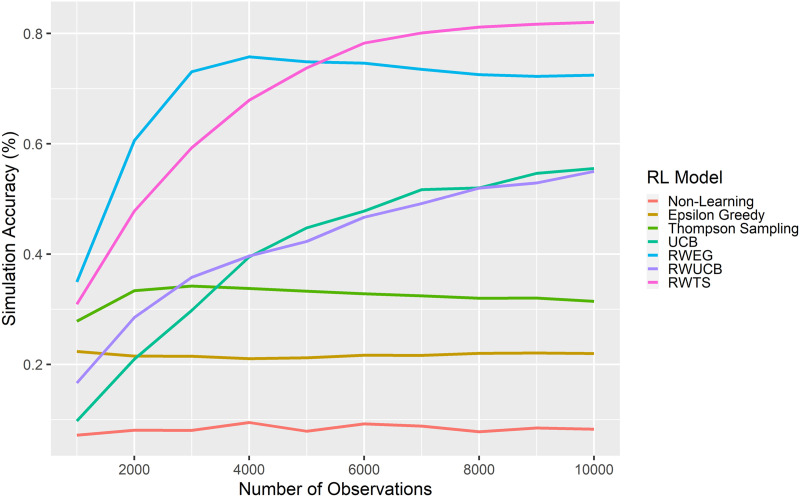
**Performance of reinforcement learning models in reproducing full-information mate choices.** *Note*. UCB = upper confidence bound; RWEG = reciprocity-weighted epsilon-greedy; RWUCB = reciprocity-weighted upper confidence bound; RWTS = reciprocity-weighted Thompson sampling.

Under the assumption that a good mate choice model should be able to approximate the decisions an agent would make under full-information conditions, I computed the proportion of the full-information model’s mate choices that each reinforcement learning model successfully reproduced. A model was recorded as having accurately reproduced the full-information model’s decisions if the agent ended up in the same end state (i.e., paired to the same partner or single if the agent never paired) as in the full-information model. Figure 2 shows performance across models and across total observation numbers. The baseline, non-learning model performed very poorly in the partial-information context, reproducing on average just *M* = 8.26% of agent decisions from the full-information context. The mate-value-as-reward reinforcement learning models performed similarly poorly, with the partial exception being the UCB model. This model did ultimately reproduce *M* = 55.52% of the full-information model’s decisions; this was higher than any of the other mate-value-as-reward models, but was also lower than the best performing models even after 10,000 observation opportunities.

In contrast, the reciprocity-weighted reinforcement models performed relatively well in reproducing the full information-model’s choices. The RWUCB model performed the worst among these, with final performance (*M* = 55.00%) and trajectory comparable to the standard UCB model. The RWEG model performed strongest at first, rising to a peak reproduction rate of *M* = 75.78% after just 4,000 observations. However, its performance plateaued at this point, dropping to an accuracy of 72.45% at 10,000 observations. Although it took longer to reach its peak than the RWEG model, the RWTS model was comparable to the RWEG model after only 5,000 observations (*M* = 73.76%). At 6,000 observations, the RWTS’s performance exceeded the peak performance of all other models and its accuracy continued to climb, reaching its plateau and the highest accuracy of all models with *M* = 82.04% at 10,000 observations.

Overall, these results indicate that reinforcement learning algorithms, especially the reciprocity-weighted models and especially the reciprocity-weighted Thompson sampling model, can be used to successfully solve the exploration-exploitation trade-off inherent to mate search even in search environments where potential mates cannot be perceived perfectly.

### Couple Simulation Proof-of-Concept

Next, I sought to determine whether the couple simulation method could successfully identify whether mate search in a population is guided by a reinforcement learning algorithm. Ordinarily, couple simulation would be applied to compare multiple models to one another—for instance, comparing reciprocity-weighted UCB to reciprocity-weighted Thompson sampling—on the assumption that different choice models systematically make different mate choices, so a given model should tend to be better at reproducing its own choices than would a different model. However, the reciprocity-weighted reinforcement learning models tested here do not differ in how they make choices: they all allocate observation opportunities to the highest mate value partners, weighted by their reciprocal interest. Rather, they differ in how they form their estimates of the mate value of potential mates. Thus, while some models are more efficient at forming accurate estimates of mate value, preliminary modeling indicated that given enough time, all models tend to converge on similar mate value estimates and thus tend to converge on largely similar mate choice predictions. For this reason, couple simulation would not be as useful in distinguishing reinforcement learning algorithms from one another. However, it could still be useful for identifying whether mate search is governed by a reinforcement learning algorithm at all. Given that the reciprocity-weighted Thompson sampling algorithm achieved the greatest observed accuracy in reproducing full-information choices and did so without demanding much more time than the other models, I proceeded with this algorithm as the focal reinforcement learning model of mate search. However, it should be noted that a reciprocity-weighted epsilon greedy model would make largely similar mate choice predictions.

**Figure 3.**
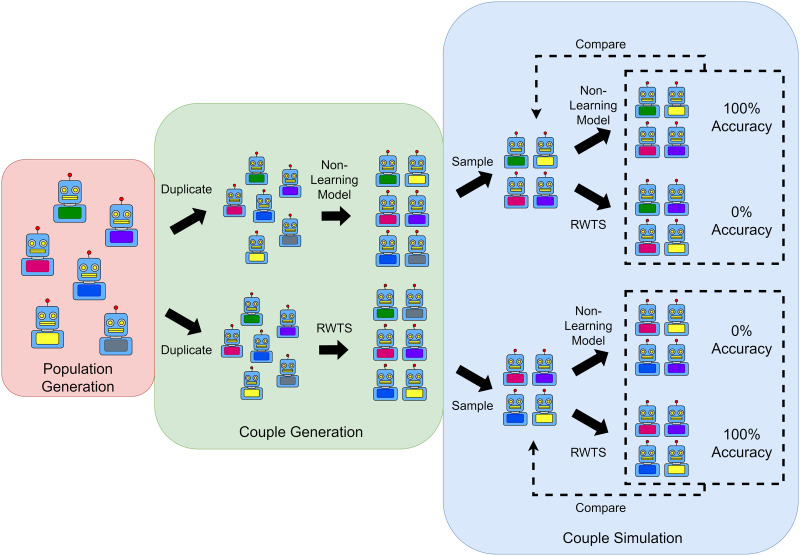
**Diagram of the couple simulation proof-of-concept model.** *Note*. RWTS = Reciprocity Weighted Thompson Sampling.

To evaluate couple simulation’s ability to identify a reinforcement learning search algorithm, I generated two identical populations of agents. I simulated these two agent populations pairing according to either a baseline non-learning model or a reciprocity-weighted Thompson sampling model, drew random samples from the resulting couples, and applied the couple simulation method to these samples to determine whether couple simulation could be used to successfully identify from which population the couples were drawn. Figure 3 diagrams this process.

#### Generating Agent Population.

I first generated an initial population of agents using the same procedure as for the model selection process. However, here I generated *N* = 50,000 agents randomly divided into 50 groups of 500 agents each. Dividing agents into subpopulations simulates sampling couples from non-overlapping mating markets.

#### Generating Agent Couples.

This agent population was next used to generate populations of agent couples that differed only in the mate search model used to form the pairs. To accomplish this, I produced two copies of the original agent population and simulated mating markets within each of these populations. In both populations, agents selected mates only within their subpopulation of *n* = 500 agents.

Mate choice proceeded nearly identically to the model selection models. Agents had 7,500 observation opportunities that they could allocate across potential mates—equivalent to 30 opportunities to observe each potential mate in their subpopulation. Each observation of a potential mate gave the agent a draw from a random normal distribution centered on that mate’s true mate value with standard deviation scaled such that 50% of the variance in mate value perceptions was due to random noise.

In one population, agents selected each other using the non-learning model. These agents observed each potential mate one time and then selected one another as mates using the resource allocation model. In the other population, all agents selected each other as mates using the reciprocity-weighted Thompson sampling model with a starting *SD* of the prior distribution set arbitrarily to *SD* = 0.10. This resulted in two populations of couples that were identical except in the model of mate search used to generate them.

#### Couple Simulation.

To assess the ability of couple simulation to identify a population’s true model of mate choice, I next drew 100 samples of size *n* = 500 from each of the two couple populations. On each sample, I simulated mate choice among the sampled agents using both the non-learning model and the reciprocity-weighted Thompson sampling model, parameterized identically as for generating the couple populations. I recorded “simulation accuracy” as the recovery rate: the percentage of original couples accurately reproduced by each model in each sample.

#### Results.

**Figure 4.**
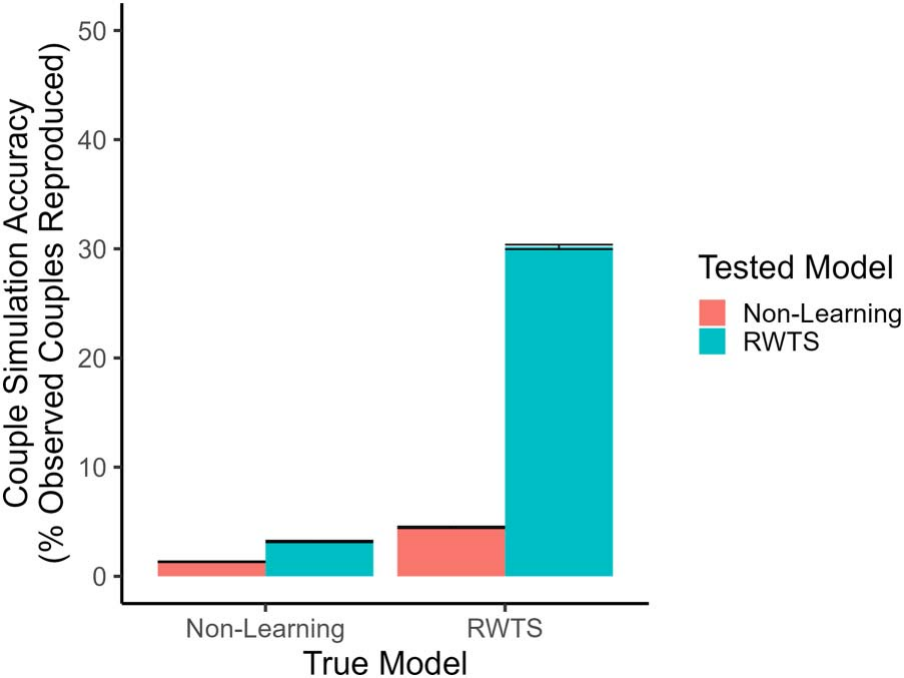
**Results of the couple simulation proof-of-concept model.** *Note*. Error bars represent 95% confidence intervals.

Figure 4 shows simulation accuracy as a function of the true model across the two couple populations. When the true model of mate choice was the non-learning model, both models of mate choice performed very poorly in reproducing sampled couples (non-learning: 1.33%, 95% CI [1.29%, 1.37%]; RWTS: 3.19%, 95% CI [3.11%, 3.26%]). However, when the reciprocity-weighted Thompson sampling model was the true model of mate choice, RWTS achieved a relatively high level of simulation accuracy (30.17%, 95% CI [29.96%, 30.38%]) whereas the non-learning model still achieved very low simulation accuracy (4.48%, 95% CI [4.41%, 4.56%]). The accuracy of the RWTS model is relatively low in this context compared to the initial model selection context because couple simulation is performed on a random sample of couples that is not identical to any of the couples’ original mating market. Some agents fail to re-pair with their original partner because they or their partners genuinely have better options available to them in this sample than they did in their original market.

Nonetheless, the RWTS model is successful at reproducing sampled couples when RWTS was the population’s true model of mate choice. This indicates that couple simulation can be used to distinguish between these two models of mate search. If mate search in a population is in reality governed by a reinforcement learning algorithm like the reciprocity-weighted Thompson sampling model, reinforcement learning models will reproduce a relatively large proportion of sampled couples in couple simulation even when perception of potential mates is highly noisy. However, if mate choice is governed by a non-learning model, couple simulation will fail to reproduce sampled couples regardless of the model tested.

Given that these models establish both that (1) reinforcement learning algorithms can successfully guide mate choice even when perceptions of potential mates a very noisy and (2) couple simulation can identify whether a population uses a reinforcement learning algorithm, I proceeded to test the performance of the reciprocity-weighted Thompson sampling model in reproducing real-world mate choices in a sample of romantically paired human participants.

## HUMAN DATA

### Methods

#### Participants.

Participants were *n* = 1,044 people from *k* = 522 committed, romantic dyads. Participants were recruited using Qualtrics’s survey panel service. Participants were *M* = 56.89 years old on average (*SD* = 13.95), ranging from 21 to 89 (*IQR* = 46, 68). Participants were in their relationships for *Mdn* = 27.83 years (*IQR* = 15, 40); relationship lengths ranged from 1 years to 64 years. Most participants described themselves as married (n = 977, 93.58%), followed by dating seriously (n = 34, 3.26%), and dating casually (n = 14, 1.34%). Nineteen participants opted to write in a separate description of their relationship status, these included “cohabitation”, “been together 15 years not married”, “committed relationship”, “divorced but together”, “domestic partner”, “living together”, “partner”, and two declined to report as described above. The partners of three participants who self-identified as “married” opted to write in a relationship status but left their status blank, resulting in an odd number of self-identified married participants.

#### Materials.

Participants reported their ideal mate preferences in a long-term romantic partner using a 31-item mate preferences questionnaire validated in Conroy-Beam et al. (2022). This questionnaire asks participants to report their ideal (i.e., most preferred) value of each 16 traits in a potential long-term, committed, romantic partner: age, affectionateness, ambition, artistic ability, disposition, family support, health, humor, intelligence, kindness, parenting, physical attractiveness, religiosity, sexiness, and status. All items except for age were represented by two items which were averaged together into a preference composite. Participants rated these items on an 11-point Likert scale ranging from extremely low on that characteristic to extremely high on that characteristic with “Average” as the midpoint. Each scale point was also labeled with a relative standing ranging from “Bottom 1 out of 100 people” to “Top 1 out of 100 people;” the relative frequency values were chosen to correspond to the percentiles associated with 11 equal steps across a normal distribution from the bottom and top first percentiles. Age was rated on an 11-point Likert scale ranging from “Under 20” to “Over 75” with 6-year age bins in between. Participants additionally rated themselves and their romantic partners on these same items using the same scale, except for age which was self-reported in years.

#### Procedure.

Participants completed ideal, self, and partner ratings in random order. Each trait rating was additionally randomized within each block. Participants completed these ratings alongside a battery of other measures not used for the analyses reported here. This sample was previously reported on in Conroy-Beam et al. (2022). All data and analysis code used for this study is available on the Open Science Framework (https://osf.io/9s24v/?view_only=d4facf0dabb5411db0012c2ccb722fc8).

### Results

The true mate values of each agent to each opposite-sex agent were calculated as the Euclidean distance between each agent’s preference vector and each potential mate’s trait vector. Agents were given 15,660 opportunities to observe potential mates, equivalent to 30 observations of each potential mate. Each observation gave the agent a signal of the potential mate’s true mate value plus random, normally distributed noise. The noise distributions were centered on zero and had standard deviation scaled such that either 0.1%, 25%, 50%, or 75% of the variance in mate value perceptions across potential mates came from noise rather than true mate value variation. These values were chosen somewhat arbitrarily to represent a range of mate value perceivability, ranging from highly veridical perception to highly noisy perception. Agents allocated observations according to ether the non-learning or the reciprocity-weighted Thompson sampling models. The standard deviation of the RWTS agents’ initial prior distributions was set to *SD* = 0.1.

**Figure 5.**
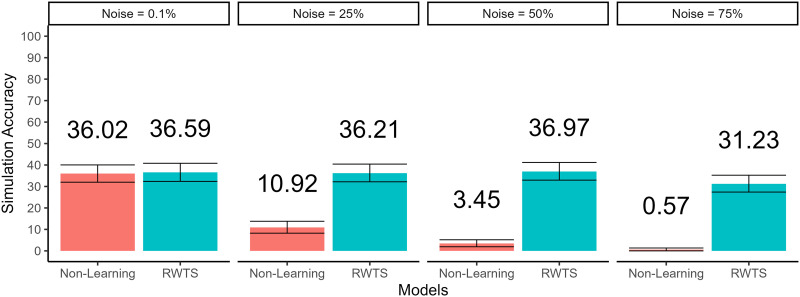
**Performance of the non-learning and reciprocity-weighted thompson sampling models in reproducing real relationships.** *Note*. Performance of the non-learning and reciprocity-weighted Thompson sampling (RWTS) models in reproducing real human relationships across different levels of assumed perceptual noise. Error bars represent bootstrapped 95% confidence intervals.

Figure 5 shows the results of couple simulation across levels of noise. When the assumed level of noise in mate value perceptions is low, the non-learning and RWTS models perform comparably well, with the non-learning model producing 36.02% couples, 95% CI [31.99%, 40.23%] and the RWTS model reproducing 36.59%, 95% CI [32.38%, 40.80%]. However, as the amount of noise added to perception increases, the performance of the non-learning model rapidly degrades. Even at just 25% perceptual noise, the non-learning model successfully reproduces just 10.92% of couples, 95% CI [8.24%, 13.60%]; at 75% perceptual noise, the non-learning model reproduces just 3 couples, yielding a simulation accuracy of 0.57%, 95% CI [0.00%, 1.24%].

In contrast, the RWTS model maintained high simulation accuracy performance even at high levels of noise. Even when 50% of the variance in perceptions came from noise, the simulation accuracy of the RWTS model still reproduced 36.97% of couples, 95% CI [32.76%, 41.19%], nearly identical to performance at baseline. At 75% noise, performance decreased slightly but was still relatively high, reproducing 31.23% of couples, 95% CI [27.39%, 35.25%].

Overall, the RWTS model performed well at reproducing the sampled couples even when mate value perception was very noisy. In contrast, the non-learning model only performed well when mate value was assumed to be very accurately perceived. This is consistent with the proof-of-concept populations in which ground-truth mate search was based on the RWTS model and is not consistent with the populations in which the non-learning model was the true model of mate choice.

## DISCUSSION

Mate choice requires choosing among an array of potential partners who, by virtue of variability in their physical, psychological, and social characteristics, vary in their suitability as mates. However, information on any given potential mate’s suitability can be gathered only in noisy samples. Mate choice consequently poses a classic exploitation/exploration trade-off: a hopeful romantic must explore their options enough to learn which mates are relatively high in value, but in order to form a relationship, eventually has to “exploit” by dedicating pursuit to those partners highest in apparent mate value. This fundamental problem of human mate choice has gone relatively unexplored despite the popularity of human mating in the anthropological, economic, psychological, and sociological literatures.

Here I begin to address this problem by modeling mate choice as a multi-armed bandit problem, where potential mates are arms of a bandit who give noisy signals of their true mate value each time they are “pulled.” An advantage of this framework is that it allows repurposing of established reinforcement learning algorithms as models of mate search and choice. Using a couple simulation approach, results indicate that mate choice is well-modeled by a reciprocity-weighted Thompson Sampling model. This model solves the exploration/exploitation trade-off by allocating pursuit opportunities in a Bayesian fashion according to a reward function that attempts to jointly maximize the perceived mate value and reciprocal interest of potential mates.

This has several implications for theories of human mating psychology. First and most directly, it provides some insight into the possible decision processes underlying mate search—an underexplored but critical aspect of the mate search process. Second, these results suggest that mate search can be successful even when the information about potential mates is extremely noisy as long as mate search algorithms appropriately balance exploration and exploitation in mate pursuit.

Third, these findings have implications for how we understand person perception in a mating context. A major set of questions in relationships research is to what extent people are able to accurately perceive themselves (e.g., Epley & Whitchurch, 2008) or their partners (e.g., Murray et al., 1996). Studies in this area typically involve comparing a single measurement of a self or partner (e.g., a rating on a Likert scale or selection of a photo from an array of modified photos) to a single measurement of a “ground truth” reality (e.g., a rating provided by the target or an unmodified photo of the target). While these approaches provide useful simplifications, reality is not as static as a single measurement: no one is equally kind across all moments and even a single photo does not perfectly capture the range of ways a person can look. We exist and experience our partners as multivariate probability distributions over our features. The reciprocity-weighted Thompson sampling model mirrors this reality in that representations of potential mates are stored not as point estimates but as probability distributions over the estimand. The strong performance of this model in reproducing real-world mate choice suggests that a fuller understanding of person perception will require approaches that, rather than relying on point estimates, focus on estimating the parameters of our perceptual distributions.

Fourth, these results offer important suggestions to researchers attempting to understand or assist real-world mate selection. For instance, many have been perplexed by the tendency of people in real-world mate choice contexts to pursue partners who do not match their preferences as measured by researchers (Eastwick & Finkel, 2008; Kurzban & Weeden, 2007; Li & Meltzer, 2015; Todd et al., 2007). For instance, in speed dating contexts, people often fail to pursue partners who match their stated preferences and instead pursue partners who do not have the qualities they desire. From the perspective of researchers, who have been able to objectively measure the characteristics of potential mates, this is apparently irrational behavior and has been interpreted by some to indicate that people may not have insight into their desires (e.g., Todd et al., 2007). From the perspective of mate seekers, though, these preference-discordant choices might represent rational exploration in the face of uncertainty. A mate seeker that always and only pursued potential mates who initially appeared to match their preferences would miss out on potential partners who make bad first impressions but ultimately turn out to be good partners. Our results suggest those interested in studying real-world mate choice behavior, or interested in providing dating recommendations, should allow participants multiple opportunities to sample potential mates to form accurate impressions rather than relying on one-shot dating decisions. How much exploration is optimal in these real-world situations, and consequently how many observation opportunities are required to detect rational choice behavior, is an empirical question left open by this work.

These models leave open several other avenues for fruitful future research. First, for example, for simplicity, these models treat mate choice as though it were a stationary learning problem. That is, mate values are assumed to be static over time. But of course, in reality, the mate value of a given mate does change over time both as a person’s own preferences change and a potential mate’s features change over time. People age, their careers develop, and their personalities and circumstances change, and these change their value to others as potential mates. Real-world mate choice is thus closer to a non-stationary bandit problem. While non-stationary bandit models could be accommodated in the couple simulation framework used here, testing them would require longitudinal mate search data. Modeling mate search more accurately as a non-stationary bandit problem would be a valuable future step toward making more realistic and detailed models of mate search.

Furthermore, here I modeled learning about potential mates at a relatively abstract level: agents receive noisy information about a potential mate’s mate value directly. This is tantamount to assuming that all information about potential mates is learned at the same rate. But in reality, some traits are more easily and rapidly perceivable than others (Miller & Todd, 1998). Physical attractiveness, for instance, can be gleaned relatively early on and accurately whereas other traits like kindness, intelligence, or financial skills take longer to accurately assess. With a model of the sequence of trait perception, one could build a comparable reinforcement learning model in which agents learn information about potential mates differentially by trait, which would more accurately reflect the true task of mate search.

Additionally, while agents in these models were uncertain about the true mate value of any particular mate, they had certain knowledge about the size and composition of their mating market. This is an unrealistic simplification. In the real world, mate seekers have a pool of potential mates from whom they could select but also know that they could find new, potentially superior potential mates by searching outside of their current pool. This poses a higher-order exploration-exploitation trade-off: one must explore enough to build a suitable pool of potential mates but ultimately must dedicate one’s efforts to pursuing known options. This search problem has previously been modeled using as analogous to the secretary problem (Todd & Miller, 1999). This has been fruitful, but requires its own unrealistic simplifying assumptions (e.g., one-shot mating decisions, no returns to previously observed partners). However, this higher-order trade-off could be accommodated within the reinforcement learning framework advanced here if agents began the model with knowledge of only a restricted set of potential mates and had to spend observation opportunities to expand their pool. This would result in both a more realistic model of the true decision problems of human mate choice and yield a unified model of the decision processes by which mating psychology solves these problems. This is a particularly important avenue for future research.

Moreover, while the proof-of-concept models suggest that couple simulation can identify whether mate search is guided by a reinforcement learning algorithm, for several reasons the results here cannot definitely support reciprocity-weighted Thompson sampling *per se* as the search algorithm that people actually use. First, couple simulation evaluates models only on their ability to reproduce real-world choices. However, the reinforcement learning algorithms tested here differ less in the choices that they ultimately predict and more in terms of the decision processes and resources used to make those choices. Finer-grained comparisons among alternative reinforcement learning algorithms would be better facilitated by behavioral tasks that allow real-time assessment of decision processes in mate search. While this would be hard to collect for real-world mate choice, behavioral evidence could come from simplified laboratory tasks including either hypothetical, computer-based search or laboratory dating events (e.g., such as the choice task in Sugawara & Katahira, 2022). In these more controlled settings, researchers could observe the search strategies people use directly and compare them to expectations from different reinforcement learning algorithms. Such behavioral evidence would provide a valuable complement to modeling data.

Furthermore, I tested here only a subset of relatively classic solutions to the multi-armed bandit problem. However, this is not an exhaustive set of possible search algorithms. There exist variants of the models tested here and models can be combined with one another, sometimes yielding superior performance (Gershman, 2018). It is also possible that one could contrive simpler heuristics that approximate or exceed the performance of the reinforcement learning algorithms considered here using simpler calculations (Gigerenzer & Gaissmaier, 2011). Future studies could compare a more exhaustive set of models to identify other plausible candidates for human mate search algorithms.

Finally, a limitation of the couple simulation approach in the way it was applied here is that it is inherently retrospective. Couple simulation was applied to reproduce relationships that already exist and existed for some time. This poses risks that can pull in two directions. One the one hand, participants may, to some extent, adjust their preferences to match the partners they have chosen (Gerlach et al., 2019). While this generally does not fully explain modeling results (Conroy-Beam, 2021), this could distort estimates of model performance. On the other hand, older participants who have been in their relationships for some time also change in both their preferences and traits from the time that they made their original mate choice. Consequently, a choice that was once optimal for them may no longer be. Consistent with this, prior work has found that newer relationships are, all else equal, somewhat easier to reproduce than more established relationships such as the ones analyzed here (Conroy-Beam et al., 2022). This suggests couple simulation may be underperforming to some extent for some of our participants. A superior approach that would avoid both of these limitations entirely would be to apply couple simulation in a prospective format. That is, one could measure the traits and preferences of single people and follow up after they have made mate choices. This would allow modeling mate search using preferences that are unaffected by partner characteristics.

In sum, mate choice requires solving a core judgment under uncertainty problem. Successful mate choice requires choosing partners with desirable features, but the features of potential mates are uncertain and revealed only noisily over time. Furthermore, time spent observing one potential mate is time that could have been spent exploring potentially superior alternatives. Here I show that this problem can be modeled in a reinforcement learning framework as a multi-armed bandit model. Agent-based models illustrate that reinforcement models, such as the reciprocity-weighted Thompson sampling model, are well-suited to solving the exploration-exploitation problem inherent to human mate choice. Furthermore, couple simulation further establishes that this reciprocity-weighted Thompson sampling model performs well in reproducing real human mate choices even under very noisy conditions. This suggests that people use reinforcement learning-like algorithms to search among uncertain options in mate choice, providing new insight into the surprisingly understudied psychology of human mate search.

## DATA AVAILABILITY STATEMENT

All data and analysis code used for this study is available on the Open Science Framework (https://osf.io/9s24v/?view_only=d4facf0dabb5411db0012c2ccb722fc8).

## FUNDING INFORMATION

This material is based upon work supported by the National Science Foundation under Grant No. 1845586.
